# Genomic Prediction of Additive and Dominant Effects on Wool and Blood Traits in Alpine Merino Sheep

**DOI:** 10.3389/fvets.2020.573692

**Published:** 2020-11-11

**Authors:** Shaohua Zhu, Hongchang Zhao, Mei Han, Chao Yuan, Tingting Guo, Jianbin Liu, Yaojing Yue, Guoyan Qiao, Tianxiang Wang, Fanwen Li, Shuangbao Gun, Bohui Yang

**Affiliations:** ^1^College of Animal Science and Technology, Gansu Agricultural University, Lanzhou, China; ^2^Animal Science Department, Lanzhou Institute of Husbandry and Pharmaceutical Sciences, Chinese Academy of Agricultural Sciences, Lanzhou, China; ^3^Gansu Provincial Sheep Breeding Technology Extension Station, Sunan, China

**Keywords:** Alpine Merino sheep, additive effects, dominant effects, prediction accuracy, genomic prediction

## Abstract

Dominant genetic effects may provide a critical contribution to the total genetic variation of quantitative and complex traits. However, investigations of genome-wide markers to study the genomic prediction (GP) and genetic mechanisms of complex traits generally ignore dominant genetic effects. The increasing availability of genomic datasets and the potential benefits of the inclusion of non-additive genetic effects in GP have recently renewed attention to incorporation of these effects in genomic prediction models. In the present study, data from 498 genotyped Alpine Merino sheep were adopted to estimate the additive and dominant genetic effects of 9 wool and blood traits via two linear models: (1) an additive effect model (MAG) and (2) a model that included both additive and dominant genetic effects (MADG). Moreover, a method of 5-fold cross validation was used to evaluate the capability of GP in the two different models. The results of variance component estimates for each trait suggested that for fleece extension rate (73%), red blood cell count (28%), and hematocrit (25%), a large component of phenotypic variation was explained by dominant genetic effects. The results of cross validation demonstrated that the MADG model, comprising additive and dominant genetic effects, did not display an apparent advantage over the MAG model that included only additive genetic effects, i.e., the model that included dominant genetic effects did not improve the capability for prediction of the genomic model. Consequently, inclusion of dominant effects in the GP model may not be beneficial for wool and blood traits in the population of Alpine Merino sheep.

## Introduction

In classical models of quantitative or complex trait genetics, the phenotypic value of each trait is controlled by a large number of loci; moreover, the interaction and alternative splicing of genes also play an extremely essential role (1). The phenotypic value is also affected by non-genetic and environmental factors (2). Therefore, it is not possible to select the top-quality animal and plant population by genotype on a single marker (or gene) or through simply observing their extrinsic features. The selection is based on the predicted total effect of the loci within an individual or their estimated breeding values (BVs) (2). Genomic selection (GS) adopts markers covering the entire genome, so that these markers can be used to explain all genetic variations (3). Compared with traditional selection methods, it has higher prediction accuracy; in addition, it could reduce the generation interval and increase the genetic progress (4, 5).

GS has improved modern breeding programs and has made a great contribution to improving the accuracy of BVs (6, 7), especially for the early selection of young individual domesticated animals (8). Genetic effects including additive effects and non-additive effects are both important for analyzing the genetic mechanism of livestock complex traits through whole-genome markers or for GP (9). However, in production and application, more attention is generally paid to additive effects, because additive genetic effects reflect the breeding value of individuals (10). Although the studies on non-additive effects has made progress in dairy cattle and beef cattle (11, 12), due to incomplete pedigrees or unreliable records, restricted calculations, and other problems, few studies have focused on the genetic, non-additive effects for sheep populations (13). The dominant effect represents interactions at the same locus; the epistatic effects represent interactions between different loci. Previous studies have suggested that non-additive genetic effects could provide an essential influence on the total variation of complex traits (14–16).

With the publication of the whole-genome sequence of dairy cows and the continuous upgrading of commercial SNP microarrays, GP and GS have been adopted on a large scale in conventional breeding programs of dairy cows (17, 18). This has also promoted the development of SNP microarrays for a variety of other livestock. To date, the associated GS technology has been gradually expanded to livestock such as beef cattle (19), pigs (20), goats (21), and sheep (22), but their development still lags far behind that of dairy cattle. The Alpine Merino sheep, the subject of the present study, possesses a mixture of Australian Merino and Tibetan sheep ancestry. They quickly adapted to the cold Qinghai-Tibet Plateau and have subsisted in high-altitude and hypoxic environments for generations. However, since the technology of GS has not been popularized in sheep, the Alpine Merino sheep population is still during the traditional phenotypic selection period. Therefore, genomic analysis to identify wool traits associated with cold tolerance and erythrocyte traits associated with adaptation to high-altitude hypoxia, critical for the selection and breeding of this population, has been used. To the best of our knowledge, GP or GS studies of Alpine Merino sheep have not so far been reported. With the availability of SNP genotypes, it is possible to explore the additive and dominant genetic effects of marker loci and thus estimate the genetic effects of each marker (23). Furthermore, in a manner similar to the construction of a genome additive relationship matrix, a genomic dominant effect matrix could also be integrated into a genomic best linear unbiased prediction (GBLUP) model for genomic analysis research (24, 25).

For this study, components of the genetic variance of the Alpine Merino sheep dataset were estimated, including additive and dominant genetic variance, then the accuracy of the BVs estimated by two models compared using cross validation. The main objective of this study was to explore the impact of additive and dominant genetic effects on the accuracy of GP and the optimization methods of GP for the Alpine Merino sheep population.

## Materials and Methods

### Ethics Statement

All animal work conducted in the present study was performed in accordance with the guidelines for the care and use of laboratory animals promulgated by the State Council of the People's Republic of China. This research was approved by the Animal Management and Ethics Committee of Lanzhou Institute of Animal Husbandry and Veterinary Medicine, Chinese Academy of Agricultural Sciences (license number: 2019-008).

### Phenotypic Data Collection

The initial phenotypic dataset was derived from animals on the HuangCheng pasture in Gansu Province, China, part of the Sheep Breeding Technology Extension Station. The dataset consisted of 11,200 individuals and included their dates of birth, region (defined by herd), and sex. In the present study, the phenotypes of 498 offspring for 26 breeding rams were collected from 7 different herds, consisting of 295 rams and 203 ewes. Most of them were half-sibs, and very few (<12%) were full-sibs, the age of which were around 14 months. A blood sample (5 mL) from each sheep was collected from the jugular vein and immediately transferred to a vacuum blood collection tube (Yuli Medical Equipment Company Ltd., Jiangsu Province, China). A standard set of red blood cell data was recorded using an H-100IV diff whole blood analyzer (Sysmex, Kobe, Japan) within 24 h of sample collection, after which the remaining blood sample was stored at −20°C (26). Four erythrocyte parameters closely associated with adaptation to high-altitude hypoxia were selected as blood trait phenotypic data, including red blood cell count (RBC), hematocrit (HCT), mean cell hemoglobin (MCH), and mean cell hemoglobin concentration (MCHC). Every year in July, when the lambs grow to 14 months of age, they reach the period of shearing. According to the agricultural industry standard of the People's Republic of China (No. NY/T 1236-2006), a 150–200-g sample of wool was collected from the abdomen of each sheep, weighed, and stored in Ziplock bags. Within 5 days of sample collection, they were sent to the National Animal and Rural Ministry of Animal and Fur Quality Supervision and Inspection Center (Lanzhou, China) for weighing, cleaning, and quality testing. Five parameters were selected to represent the phenotypic data of wool traits, including staple length (SL), clean fleece weight rate (CFWR), mean fiber diameter (FD), fleece breaking strength (FBS), and fleece extension rate (FER). Supplementary Table 1 shows the detailed definition of the above wool traits parameters.

### Genotyping and Quality Control

In total, 498 Alpine Merino sheep were genotyped using a custom Affymetrix HD 630K microarray. The genotyping platform adopted for analysis was based on the GeneTitan System (Santa Clara, CA, USA) Array Plate Processing Workflow from Thermo Fisher (Affymetrix). Prior to statistical analysis, SNPs were pre-processed using PLINK v1.9b4 software (27). Samples were eliminated where SNPs had a call rate lower than 95%, a Hardy–Weinberg equilibrium *P* < 10e-6, a minor allele frequency <0.01, and those with more than 10% of their genotype missing. Moreover, in order to eliminate the potential biased analysis caused by gender and maternal effects, the X, Y, and mitochondrial chromosomes were excluded from analysis (11). Following filtering and quality control, a total of 498 individual animals with 441,740 autosomal SNPs were retained.

### Statistical Methods for GP

In this study, we explored the possibility of combining both additive and dominant genetic effects in the genomic evaluation and further compared the accuracy of GP via different models. Nine traits of 498 samples were used in (1) estimating the variance of components in the genotype dataset of Alpine Merino sheep, including additive and dominant variance, and (2) analyzing the accuracy of GP using 5-fold cross validation and then comparing the accuracy of estimation of BVs between the prediction model using additive effects and that when combining both additive and dominant effects. Both models were evaluated within a GBLUP framework. Replicate measurements were not available for the sheep; thus, permanent environmental effects were not modeled. Samples were of different genders and from different herds, factors that altered phenotypes in a fixed manner, so system environmental effects were added to the framework.

The components of variance of additive genetic effect ($\sigma_{A}^{2}$) and residual effect ($\sigma_{E}^{2}$) were estimated adopting the MAG model.

(1) $$\begin{array}{l} y=Xb+Zu+e \end{array}$$

where ***y*** represents the phenotypic value of the individual and *b* refers to the vector of fixed effects, since the individuals involved in the current study were from different herds; the fixed effects include the gender (male and female) of each individual and the different herds (H1–H7). *u* is the vector of breeding values of the individual, ***e*** is the vector of residual effects, ***X*** is the design matrix corresponding to fixed effects, and ***Z*** is the design matrix corresponding to breeding values. Covariance matrices of additive effects were $Var\left(u\right)=G\sigma_{a}^{2}$, where *G* is the matrix of the genomic relationship, calculated based on the approach of VanRaden (28) and using R package “HIBLUP” (https://github.com/xiaolei-lab/hiblup):

(2) $$\begin{array}{l} G=\frac{W_{1}W_{1}^{T}}{2\sum_{i=1}^{m}p_{i}\left(1-p_{i}\right)} \end{array}$$

where *W*_1_ is the matrix of additive genetic effect markers, with dimensions of the number of individuals (*n*) by the number of loci (*m*), and *p*_*i*_ is the minor allele frequency (MAF) value of locus *i*.

In addition, the components of variance of the additive genetic effect ($\sigma_{A}^{2}$), dominant genetic effects ($\sigma_{D}^{2}$), and residual effect ($\sigma_{E}^{2}$) were estimated using the MADG model:

(3) $$\begin{array}{l} y=Xb+Zu+Zv+e \end{array}$$

As with the MAG model, in Equation (3), ***y***, *b*, *u*, ***e***, ***X***, and ***Z*** represent the same parameters as those defined in Equation (1), while *v* refers to the vector of the dominant effect of an individual. The covariance matrices of dominant effects were $Var\left(v\right)=D\sigma_{d}^{2}$, *where D* represents the matrix of the genomic dominant relationship (25), also calculated using R package “HIBLUP” (https://github.com/xiaolei-lab/hiblup):

(4) $$\begin{array}{l} D=\frac{W_{2}W_{2}^{T}}{4\sum_{i=1}^{m}p_{i}^{2}{\left(1-p_{i}\right)}^{2}} \end{array}$$

where *W*_2_ represents the matrix of dominant genetic effect markers and where, *n*, *m*, and *p*_*i*_ are the same as those defined in Equation (2).

### Accuracy of Breeding Value Predictions by Cross-Validation

The accuracy of GP was evaluated by 5-fold cross validation. The dataset was randomly divided into 5 approximately equally sized subgroups (each subgroup contained about 100 individuals), for each cross validation, of which 4 were considered as training groups (reference population) to estimate parameters, and the remaining group (candidate population) was used to validate samples. The population was generally randomly divided when performing 5-fold cross validation. A number of validation samples had offspring in the training population, causing in that case the cross validation to be based on offspring, which would exaggerate the accuracy of the prediction (29). In the current population, individuals were collected from the same generation, ensuring that verification samples did not have offspring in the training group. In this method of grouping, the 5-fold cross validation evaluated the accuracy of ewes and rams of the same generation. Therefore, it was effective in limiting the bias in accuracy caused by offspring in the training group (11).

Based on the MAG and MADG models, the breeding values of the validation group were predicted and their respective components of variance estimated. In addition, we performed the 5-fold cross validation described above twice to ensure randomness of the verification group. Finally, 10 different values of accuracy were calculated for each trait, the mean value recorded as the final accuracy.

## Results

### Phenotypic Statistics and Genotypic Characteristics

Table 1 displays descriptive statistics of the phenotypic measurements of the sheep, including mean of each parameter, abbreviations of each trait standard error, coefficient of variation, and number of individuals. In detail, it shows five wool traits and four blood traits. For wool traits, the coefficient of variation (CV) ranged from 0.1 (staple length) to 0.3 (Fleece extension rate), and the standard error (SE) ranged from 0.1 (mean fiber diameter) to 0.4 (fleece breaking strength); for blood traits, the CV ranged from 19.9 (mean cell hemoglobin) to 29.9 (hematocrit), and the SE is ranged from 0.0 (hematocrit) to 4.7 (mean cell hemoglobin concentration).

**Table 1:** Descriptive statistics of phenotypic values of traits.

**Trait**	**Abbreviation**	**Mean ± SD^a^**	**CV^b^ (%)**	**SE^c^**	**Number**
Staple length (mm)	SL	83.5 ± 9.4	0.1	0.4	494
Clean fleece weight rate (%)	CFWR	66.1 ± 6.4	0.1	0.3	494
Mean fiber diameter (mm)	FD	21.4 ± 2.2	0.1	0.1	491
Fleece breaking strength (N/ktex)	FBS	33.3 ± 8.0	0.2	0.4	491
Fleece extension rate (%)	FER	19.5 ± 5.1	0.3	0.2	493
Red blood cell count (10^12^/L)	RBC	7.7 ± 1.7	22.1	0.1	496
Hematocrit (%)	HCT	0.3 ± 0.1	29.9	0.0	492
Mean cell hemoglobin (pg)	MCH	13.2 ± 2.6	19.9	0.1	494
Mean cell hemoglobin concentration (g/L)	MCHC	377.5 ± 103.8	27.5	4.7	489

a *SD, standard deviation*.

b *SE, standard error*.

c *CV, coefficient of variation*.

### Prediction of Breeding Values and Total Genetic Values

Estimated components of variance for the MAG and MADG models are presented in Table 2. Additive variance (showed as a proportion of total genetic variance) for wool traits ranging from 6.88% (fleece extension rate) to 49.5% (staple length) and for blood traits from 13.31% (red blood cell count) to 27.94% (mean cell hemoglobin concentration). Dominant variance (expressed as a percentage of total genetic variance) for wool traits ranged from 0.00 to 73.46%. The dominant variance of staple length and mean fiber diameter were extremely low, approximately equal to 0.00%. The dominant variance of clean fleece weight rate, fleece breaking strength, and fleece extension rate were 4.22, 10.06, and 73.41%, respectively. Dominant variance for blood traits ranged from 0.00 to 27.99%. The dominant variance of mean cell hemoglobin and mean cell hemoglobin concentration were approximately equal to 0.00%. The dominant variance of red blood cell count and hematocrit were 27.99 and 24.99%, respectively.

**Table 2:** Estimates of additive and dominant components of variance obtained using HIBLUP for MAG and MADG models.

**Trait^a^**	**MAG**			**MADG**			
	**$\sigma_{a}^{2}$(SE)**	**$\sigma_{e}^{2}$(SE)**	**$\frac{\sigma_{a}^{2}}{\sigma_{a}^{2}+\sigma_{e}^{2}}$(SE)^*b*^**	**$\sigma_{a}^{2}$(SE)**	**$\sigma_{d}^{2}$(SE)**	**$\sigma_{e}^{2}$(SE)**	**$\frac{\sigma_{a}^{2}}{\sigma_{a}^{2}+\sigma_{d}^{2}+\sigma_{e}^{2}}$(SE)^b^**
SL	40.96 (11.16)	41.79 (9.78)	0.50 (0.12)	30.25 (17.93)	0.00 (34.61)	48.81 (21.45)	0.00 (0.44)
CFWR	11.93 (4.68)	17.79 (4.24)	0.40 (0.15)	11.41 (8.54)	1.25 (17.53)	17.05 (11.27)	0.04 (0.59)
FD	1.54 (0.55)	2.61 (0.51)	0.37 (0.13)	4.11 (3.02)	0.00 (5.21)	4.43 (3.16)	0.00 (0.61)
FBS	18.03 (7.99)	35.34 (7.45)	0.34 (0.14)	15.50 (14.46)	5.36 (28.20)	32.44 (17.79)	0.10 (0.53)
FER	1.34 (1.96)	18.10 (2.22)	0.07 (0.10)	0.00 (8.11)	22.29 (12.24)	8.07 (6.98)	0.73 (0.40)
RBC	0.35 (0.31)	2.25 (0.33)	0.13 (0.12)	0.06 (0.71)	0.73 (1.67)	1.81 (1.07)	0.28 (0.64)
HCT	0.001 (0.001)	0.005 (0.008)	0.24 (0.12)	0.001 (0.001)	0.00 (0.00)	0.004 (0.002)	0.25 (0.48)
MCH	1.34 (0.78)	4.48 (0.78)	0.23 (0.13)	2.03 (2.61)	0.00 (6.10)	5.59 (3.97)	0.00 (0.81)
MCHC	2867.17 (1420.81)	7395.22 (1371.06)	0.28 (0.13)	4388.68 (4504.31)	0.00 (1001.76)	8854.82 (1428.41)	0.00 (0.76)

a *SL, staple length; CFWR, clean fleece weight rate; FD, mean fiber diameter; FBS, fleece breaking strength; FER, fleece extension rate; RBC, red blood cell count; HCT, hematocrit; MCH, mean cell hemoglobin; MCHC, mean cell hemoglobin concentration*.

b *Heritability, the ratio of the additive effect variance to the total phenotypic variance*.

**Figure 1:**
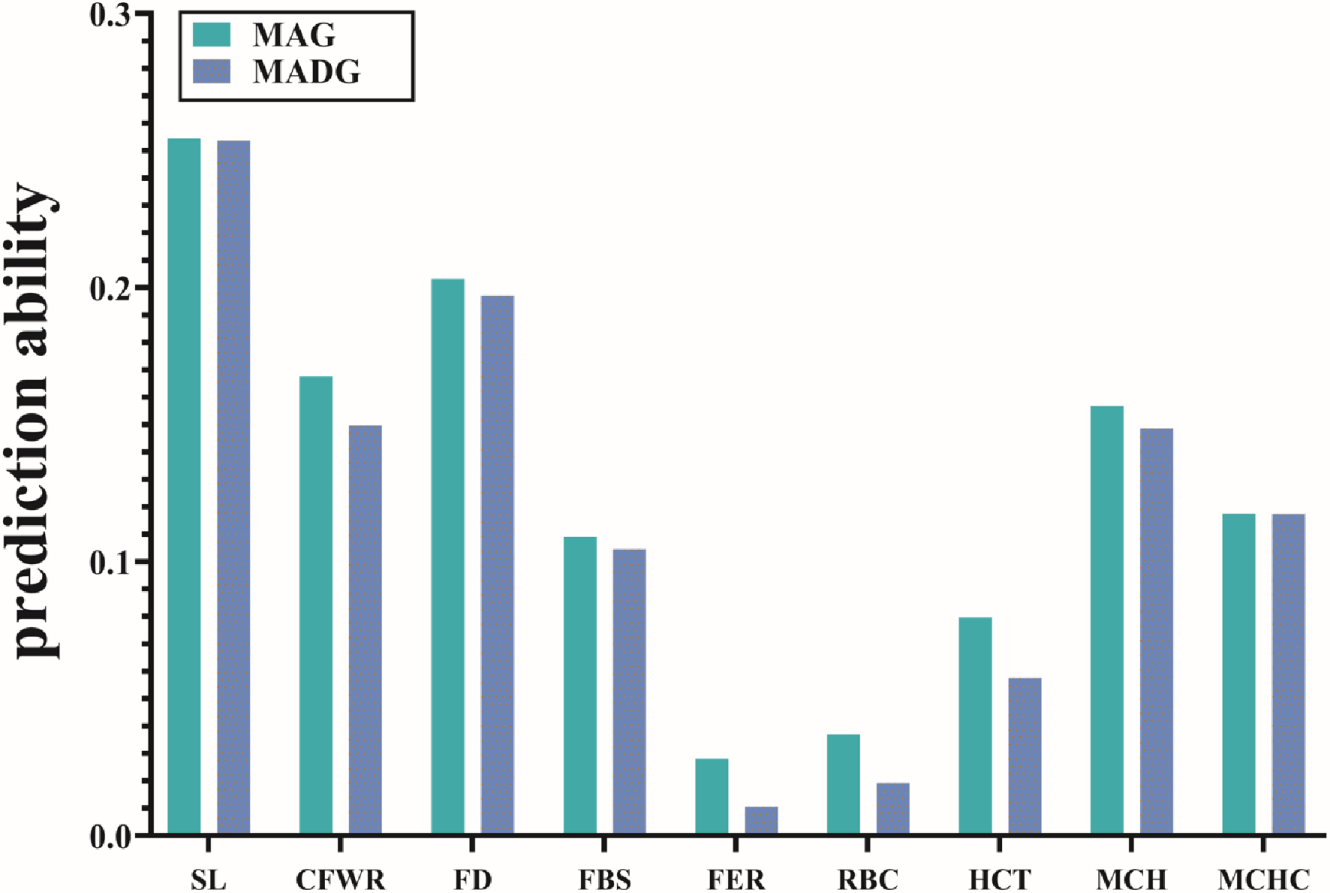
Prediction accuracies of 9 traits using the two models. Different colors represent different models. Abbreviations of traits are explained in Table 2.

Table 3 compares the capability of GP for the two models. The accuracy calculated by 5-fold cross validation varied with the different traits. For wool traits, the prediction accuracy of FER was least (MAG = 0.03, MADG = 0.01) while that of SL was greatest (MAG = 0.25, MADG = 0.25). For blood traits, the prediction accuracy for RBC was least (MAG = 0.04, MADG = 0.02), while that of MCH was greatest (MAG = 0.16, MADG = 0.15). Of all 9 traits, including wool and blood traits, FER and RBC displayed relatively low prediction accuracy (<0.1), while the others displayed low to medium accuracy (0.11–0.25). In addition, the MAG model exhibited accuracy slightly higher than that of the MADG model in all traits (Figure 1), regardless of whether heritability was high or low.

**Table 3:** Comparison of prediction accuracies of 9 traits.

**Trait^a^**	**Prediction accuracy^b^**	
	**MAG**	**MADG**
SL	0.25 (0.02)	0.25 (0.02)
CFWR	0.17 (0.03)	0.15 (0.03)
FD	0.20 (0.02)	0.20 (0.02)
FBS	0.11 (0.03)	0.10 (0.04)
FER	0.03 (0.02)	0.01 (0.03)
RBC	0.04 (0.02)	0.02 (0.04)
HCT	0.08 (0.02)	0.06 (0.03)
MCH	0.16 (0.03)	0.15 (0.03)
MCHC	0.12 (0.02)	0.12 (0.02)

a *Abbreviations of traits explained in Table 2*.

b *SE are in parenthesis*.

## Discussion

### Genome Relationship Matrix

Genome-wide dense markers have replaced pedigree information and have provided a new methodology for estimating additive and dominant genetic effects, anticipated to improve the accuracy of genomic predictions (30). The technique of construction of an additive genomic relationship matrix by whole-genome markers (28, 31) then using a linear mixed model to estimate individual breeding values has been widely used in genomic prediction and selection (32–34). In the present study, in addition to an additive genomic relationship matrix, we also constructed a dominant relationship matrix via genome-wide SNP markers. The genome relationship matrix represents confirmed gene sharing, rather than merely conceptualized or predicted ancestral sharing (24). In contrast to pedigree-based individual relationship matrices, a genomic relationship matrix based on genome-wide dense markers is able to capture genetic links from unknown common ancestors (24), not available in an individual relationship matrix based on pedigree. Furthermore, a genomic relationship matrix is suitable for not only populations with pedigree information but also those without pedigree information. It is particularly useful for the study of livestock populations and even wild populations that lack or have inaccurate pedigree information (35, 36). The technique for construction of an epistatic genome relation matrix allows for only approximate calculations when considering a large number of markers. Where epistasis between several specific markers is modeled using a required technique, an epistatic genomic relationship could only be approximated, as it is difficult to construct a precise epistatic relationship matrix (37, 38). Therefore, an epistatic relationship matrix was not constructed in the current study.

The use of an additive and dominant genetic relationship matrix in a typical linear mixed model (such as the GBLUP model) is capable of estimating additive and dominant components of variance. Goddard and Hayes et al. established that the GBLUP model is equivalent to a linear random regression model, assuming that each SNP causes an effect, and the effects of all SNP effects follow a normal distribution with equal variances (39, 40). The predictability of the GBLUP model may be unsatisfactory in cases where a few markers have large effects and the majority have little effect or are even ineffective. However, the study of actual data from German Holstein cows has demonstrated that the GBLUP model, which directly estimates genomic breeding value, was able to fully utilize the related information (41). Thus, the GBLUP model has comparative advantages in the case of close relationships between reference and candidate populations (42, 43). Considering that the population involved in the current study was from the same breed, the method proposed in the present study may not be the only one, but it was an appropriate approach for estimation of additive and dominant variances and for prediction of genomic breeding values.

Additive and dominant variance components were estimated for 9 traits of interest in the Alpine Merino sheep population, including 5 wool traits and 4 blood traits. Due to complex genetic mechanisms and differences between species, to date, few studies have estimated genetic variance components in sheep blood traits. Safari and Fogarty et al. collected and summarized a number of studies on the genetic parameters of important traits in sheep. Their reported results suggested that the weighted average heritability of FD (0.51–0.59) was based on 41 and 43 estimates, respectively; the weighted average heritability of SL (0.46–0.48) was based on 21 estimates; the weighted average heritability of FBS (0.34) was based on 11 estimates; and the weighted average heritability of CFWR (0.34–0.51) was based on 36 and 43 estimates, respectively (44, 45). In this study, the heritability of 4 wool traits (Table 2) was close to the results reported in previously published literature except that 0.37 for FD was slightly lower. In particular, SL (0.50) and FBS (0.34) were very close to the results reported in the literature. The results suggested that although the dataset was only a small proportion of the entire Alpine Merino population, heritability estimates were still reliable. For the 9 traits involved in the current study, the estimated values of dominant variance ranged from 0 to 73.41% of the total genetic variation. It has been reported in the literature that estimated variation in dominant milk production traits in Holstein cows ranged from 1.4 to 42.9% of the total genetic variance, with a wide range of variation (46–48). This is consistent with the research results in the present study, but with a smaller range. Two reasons may explain why the range of our results was larger than that reported in the literature. (1) An excellent rearing bull is able to participate in the breeding of a large number of cows through semen cryopreservation technology, hard to achieve in the Alpine Merino sheep population. In a similar-sized population, the male-to-female mating ratio of Alpine Merino sheep was considerably larger than that of Holstein cows, proving richer genetic diversity to the Alpine Merino sheep, leading to greater population heterozygosity and QTL alleles with greater intermediate frequencies (49). (2) Research reported in the literature was based on an individual relationship matrix constructed using a pedigree dataset, then a conventional BLUP method used to estimate the various components of variance, while the individual relationship matrix was constructed from genomic information in the present study, which is expected to improve the ability to estimate components of variance, including additive and dominant variance, and also effectively reduce potential confusion about additive effects and residuals. This confusion can also lead to the different estimations of components of variance (11). Previous studies have shown a significant contribution of non-additive genetic variation. It has been reported that in a Duroc pig population, the non-additive variance of the majority of meat quality and carcass traits accounts for more than 50% of total QTL variance (50). The ratios of dominant variance to additive genetic variance ranged from 15% (21-day litter weight) to 57% (interval between parities) for reproductive and growth traits in South African Duroc pigs (16). In chickens, QTL analysis demonstrated that non-additive genetic effects explained greater variation in those younger than 46 days, while additive genetic effects explained the principal proportion of later life genetic variation (51, 52). These results indicate that non-additive genetic variation is extremely important in complex traits.

### GP Results and Accuracy of Prediction

To the best of our knowledge, studies of GP or GS about high-altitude hypoxia adaptation in sheep were rarely reported, so we did not find reference statistics related to sheep blood traits. However, for wool traits, the statistical averages of FD and FBS calculated by Daetwyler et al. in their study on genetic parameter estimation of Australian Merino sheep were 17.3 and 33.4, respectively (53). The statistical averages of SL and FBS obtained by Moghaddar et al. in the genomic prediction study of wool traits of Poll Dorset (PD) and Merino and White Suffolk (WS) were 80.93–98.57 and 33.80–35.61, respectively (54); in addition, according to Hamadani and coworkers in the study of Rambouillet sheep heritability estimation, the statistical average of FD is 21.26 (55). In the current study, the statistical results (Table 1) were consistent with the values calculated from the above studies, and it suggested that the statistical values of the phenotypic measurements were reliable.

In the present study, the accuracy of GP was evaluated by 5-fold cross validation, the results of which are presented in Table 3. The results of the GP study on wool traits of different breeds of sheep from Daetwyler et al. suggested that for FD traits, the accuracy of prediction ranged from 0.23 to 0.79, and for FBS traits, it ranged from−0.01 to 0.43 (53). Moreover, Moghaddar and his colleagues estimated the BVs of wool trait such as FD, SL, and FBS of Merino, Border Leicester (BL), and WS; the prediction accuracy of FD, SL, and FBS was between 0.39 and 0.50, 0.25 and 0.61, and 0.10 and 0.49, respectively (54). In the current study, the prediction accuracy of FD, SL, and FBS was 0.20, 0.25, and 0.11 respectively, which were close to the results reported in the previous literatures. For these 9 traits, regardless of whether the MAG or MADG model was adopted, the accuracy of prediction of FER was least, while SL was correspondingly the greatest. The additive variances estimated by the two traits described above are also the smallest and the largest, respectively, at 0.07 and 0.50. A number of studies have indicated that, as the level of heritability increases, the accuracy of genome prediction also increases (39, 56, 57). This was also found in the current study: traits with high heritability, such as SL and FD, showed higher accuracy of prediction than FER and RBC, which, with low heritability, suggested that the level of additive genetic variance has a positive effect on the accuracy of prediction. Moreover, it is seen from Table 2 that the additive and dominant variances of CFWR were higher than those of FD, but with an accuracy of prediction slightly lower than that of FD (Table 3). Interestingly, this was also found in blood traits; additive and dominant variances of HCT were higher than those of MCH, but the accuracy of prediction of the former was lower. The results above suggested that the dominant variance also has a vital impact on predictability of the model. Alves and coworkers analyzed nine fertility and fecundity traits of dairy cows adopting the model containing additive effects and dominant effects and got similar conclusions: models which include non-additive genetic effects for the majority of traits indicate that the effects of epistasis, dominance, or a combination of the two are as important as the additive effect and occasionally contribute substantially more than the additive effect (8). In this study, for those traits with dominant variance, both additive and dominant variances affect the accuracy of prediction. From the comparison of CFWR and FD, it can be explained that dominant effects may have an impact on the accuracy of prediction greater than that of additive effects in some traits.

For these two models (MAG and MADG), the accuracy of prediction of the MAG model was slightly higher than that of MADG, for both wool and blood traits. The proportion of full-sib relatives and the relationship coefficient of the dominant effect between the training and verification datasets are very small because samples were selected from the same generation with different dams. The MADG model, which combined additive and dominant genetic effects, did not exhibit a clear advantage over the MAG model with only the additive genetic effect, possibly a reflection of only a small proportion of the dominant variance effect information being transferred from the training group to the verification group through 5-fold cross validation. This has also been found in previous studies: Ertl and his colleagues found by genomic analysis of Fleckvieh cattle that when the whole sib relationship between training and validation datasets is small, the accuracy of prediction of the total genetic value by cross validation is not higher in the dominant model than in the additive model (11). In the simulation study of Varona et al. (58), only the population with full-sibs or full-sib offspring could capture the change of breeding value when the additive model was converted to a dominant model (58). The samples collected were selected from the whole Alpine Merino herd in the present study and differed from the Holstein cow dataset that had a large proportion of full-sib relatives. Estimated results from full-sib offspring would not therefore represent the whole population. The results of the present study demonstrated that, compared with the MAG model, the MADG model which included the dominant effect displayed no apparent advantage in terms of predictability, not surprising since the additive genetic effects account for a proportion of the epistasis and dominant effects (8). The inclusion of non-additive genetic effects, therefore, in model fitting is not consistently advantageous (10, 13, 59), which is in agreement with the majority of previous studies (60–62).

For the accuracy of GP, the MADG model containing dominant genetic effects does not show a significant advantage over the MA model with only additive effects. Although the advantage of the MA model over MAD in these traits involved in the current study was not obvious, it could not be ignored. It could be expected that when a larger reference dataset was adopted, especially when it contains more offspring animal records, comparing the predictive ability of models with additive effects and models with additive and dominant effects will get greater benefits for the GP; meanwhile, it is not excluded that genetic models containing additive and dominant effects may be beneficial to the development of specific capabilities of integration for other important traits (34). Furthermore, a number of studies have suggested that there exists a dependent relationship between additive and dominant effects (63, 64). Although the processing of these relationships was somewhat complicated and the calculations substantial, we will collect a larger dataset and attempt to take these factors into consideration in subsequent studies.

## Conclusions

In summary, the present study estimated the additive and dominant variances of 9 traits of Alpine Merino sheep based on two different GBLUP models and used 5-fold cross validation to evaluate the accuracy of prediction of breeding values for these traits. This was the first time GP has been applied to the domesticated Alpine Merino sheep population. Dominant genetic effects account for a large proportion of total phenotypic variation in particular traits (FER, RBC, HCT). Both additive and dominant variances play a vital role in the accuracy of prediction, while for some traits the latter may have a greater impact on the accuracy of prediction than the former. In addition, this study indicates that the predictive capability was not improved when dominant effects were included in the model if the proportion of full-sib relatives in the population was small.

Based on the current results, we will expand the scale of the dataset in the subsequent study and continue to research GP by adding other important traits of the Alpine Merino sheep population, in order to provide more theoretical references for the breeding of this sheep population. Moreover, this study adopts genome-wide SNP information to construct additive and dominant relationship matrices; compared with the relationship matrix based on pedigree, the former is obviously more reliable, which suggests the great contribution of genome-wide SNP information in genome selection. Although the individuals involved in this study only included the Alpine Merino sheep population, the heritability estimation results of SL and FD and the accuracy of GP were very close to those reported in the previous literature. Therefore, it is not excluded to extend these study methods and apply them to other breeds of sheep.

## Data Availability Statement

The datasets presented in this study can be found in online repositories. The names of the repository/repositories and accession number(s) can be found at: https://figshare.com/, https://figshare.com/articles/GS_sample/12497993, https://figshare.com/articles/GSQC_bed/12497963.

## Ethics Statement

The animal study was reviewed and approved by Animal Management and Ethics Committee of Lanzhou Institute of Animal Husbandry and Veterinary Medicine, Chinese Academy of Agricultural Sciences. Written informed consent was obtained from the owners for the participation of their animals in this study.

## Author Contributions

SZ: conceptualization, formal analysis, and writing of the original draft. SZ and CY: data curation. BY: funding acquisition. HZ and MH: investigation. YY and TG: methodology. SG and BY: project administration and writing of the review and editing. TW and FL: resources. JL and CY: software. HZ: validation. GQ: visualization. All authors contributed to the article and approved the submitted version.

## Conflict of Interest

The authors declare that the research was conducted in the absence of any commercial or financial relationships that could be construed as a potential conflict of interest.
