# Supplementary material for: Genomic Prediction of Additive and Dominant Effects on Wool and Blood Traits in Alpine Merino Sheep
Source: Front Vet Sci. 2020 Nov 11;7:573692. doi: 10.3389/fvets.2020.573692 (PMC7686030; doi:10.3389/fvets.2020.573692)
Supplement: Supplementary file 1 [file Table_1.pdf]

Table S1. Definition of wool traits

Trait[1]	Related definitions
SL	The length obtained by measuring the weight along the axis of the wool tuft when the fibers in the tuft are not stretched and crimped and are not damaged
CFWR	The weight of the pure wool fiber under the condition of the public moisture regain accounts for the percentage of the weight of the original wool sample
FD	Longitudinal projection width of wool fiber
FBS	The ratio of breaking strength (the maximum force recorded when the sample is broken during the tensile test) to the fiber density
FER	Increment of sample length due to tensile force

[1] SL, staple length; CFWR, clean fleece weight rate; FD, mean fiber diameter; FBS, fleece breaking strength; FER, fleece extension rate;
